# Zinc Monotherapy as an Alternative Treatment Option for Decompensated Liver Disease due to Wilson Disease?

**DOI:** 10.1155/2020/1275940

**Published:** 2020-01-14

**Authors:** Hansa Haftu, Mohammed Mustefa, Teklu Gebrehiwot

**Affiliations:** ^1^Mekelle University, College of Health Science, Pediatric and Child Health Department, Tigray, Ethiopia; ^2^Mekelle University, College of Health Science, Clinical Pharmacist, Tigray, Ethiopia

## Abstract

**Background:**

Wilson disease is a rare metabolic disorder involving copper metabolism, and patients may present with a variable degree of hepatic, neurologic, and psychiatric manifestations. In the case of hepatic presentation, treatment is usually initiated with potentially toxic copper chelators (D-penicillamine or Trenton). Although zinc is of low toxicity and low cost for treatment of Wilson disease, it has been limited to the adjunctive as a single maintenance drug or for asymptomatic patients. The use of zinc monotherapy in patients suffering from a severe liver disease was not well studied. In our case report, we describe a pediatric patient who presented with liver failure and the use of zinc monotherapy in patients with severe hepatic manifestations. *Case presentation*. A 15-year-old male patient from Ethiopia presented with generalized body swelling (edema and ascites) with yellowish discoloration of his eyes and easy fatigability. He had hyperbilirubinemia, coagulopathy, hypoalbuminemia, and deranged liver enzymes. He had a Keyser–Fleischer ring visible with the naked eye, which was confirmed by slit-lamp examination. He had very low serum ceruloplasmin (<8 mg/L) and high 24-hour urine copper (150 mcg/dl). In accordance with the scoring system proposed by the 8th International Meeting on Wilson Disease and Menkes Disease, a diagnosis of Wilson disease was made. Zinc monotherapy with low copper diet was initiated for decompensated liver disease due to Wilson disease because of the inaccessibility of chelators (D-penicillamine or Trientine). After months of treatment with zinc, the patient experienced normalization of hepatic synthetic function and resolution of hypoalbuminemia and coagulopathy. The patient had also clinically stabilized (ascites, lower extremity swelling, edema, and jaundice were improved. Currently, the patient is on follow-up almost for the last four years in the gastrointestinal clinic.

**Conclusion:**

Our case shows that zinc has the potential for treatment in improving liver function. Though zinc has its own side effects, it is important and maybe an alternative treatment option in those with limited resources (not able to access chelators). This example hopefully will encourage future investigations and researches on zinc monotherapy for treating symptomatic decompensated hepatic Wilson disease.

## 1. Background

Wilson disease (WD) is a rare autosomal recessive genetic disorder of copper metabolism, which is a defective biliary excretion of copper, resulting in the accumulation of copper in the liver, brain, kidney, and cornea. The disease affects one in 100,000 individuals [1, 2]. Patients with WD have varying clinical presentations ranging from an asymptomatic state to acute life-threatening liver involvement (acute liver failure), which can cause high mortality reaching 95% without liver transplantation [1, 3, 4]. Other patients may present with chronic liver disease mimicking other etiologies and neuropsychiatric manifestations. Though acute liver failure (ALF) presentation is rare in pediatric age groups (only 3% of all ALF case series in children), it is dramatic and may result in suddenness [3]. Although Wilson disease is rarely diagnosed and reported in African children, it must be considered differential in any patient with liver disease. There are few reports regarding zinc monotherapy in patients with decompensated liver disease. Our case report documents the role of zinc monotherapy in clinical resolution and normalization of laboratory liver synthetic dysfunction in patients with the severe hepatic presentation of Wilson disease.

### 1.1. Case Presentation

**Figure 1:**
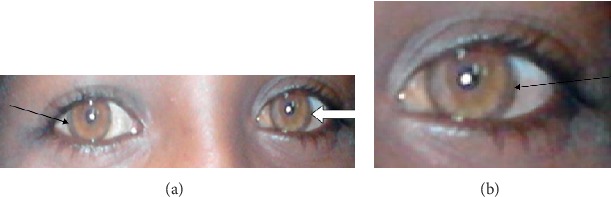
(a, b) Both arrows (black and white arrows) show the Kayser-Fleischer ring before the treatment.

A 15-year-old male child, from nonconsanguineous marriage, was referred to our hospital from a private clinic with a possible diagnosis of renal disease in December 2015. His main complaint was generalized body swelling of 3 months in duration. In the private clinic, he was given Lasix for the diagnosis of renal disease, but the symptoms got worse and he discontinued the medication. He had easy fatigability and shortness of breath at rest and loss of appetite associated with yellowish discoloration of the eye for two weeks. He was a grade seven student. He had no change in school performance, no difficulty in playing and writing, and no behavioral change or abnormal body movement. He had no history of vomiting, change in bowel habit, abdominal pain, drug, or herbal medication use, alcohol intake, previous history of jaundice or contact with a jaundiced person, and family history of similar illness. He also had no history of bleeding from any site. His developmental history was optimal. The physical examination showed that he was conscious and oriented to time, person, and place. He had icteric sclera, a well-formed Kayser–Fleischer ring visible with the naked eye (Figure 1), which was confirmed by slit-lamp examination, grossly distended abdomen with shifting dullness and fluid thrill, pitting pedal, and pretibial edema. Initial investigations showed deranged liver function (Table 1). His serum creatinine and blood urea nitrogen, serum electrolytes, lipid profile, and urine analysis were normal. A serologic test for the human immune deficiency virus (HIV), hepatitis B virus surface antigen (HBsAg), antinuclear antibody (ANA), and antibodies to the hepatitis C virus (HCV) were negative. Serum ceruloplasmin was very low, less than 8 mg/dl (normal 20–60 mg/dl), and the 24-hr urinary copper excretion was high (150 mcg/dl). Abdominal ultrasound showed that the liver size was normal, but it was coarse and nodular with ascites. For our patient, Wilson disease was diagnosed according to the scoring system proposed by the 8th International Meeting of Wilson Disease and Menkes Disease [4], with a total score of 4 (serum ceruloplasmin = 2, K-F ring = 2). The patient was admitted and started on diuretics, low protein and salt diet, bisacodyl metronidazole, multivitamins, and parental vitamin K. After Wilson disease was confirmed, the patient started on zinc acetate (neither penicillamine nor trientine was available, and even more, it was difficult to bring them from abroad) and was advised to avoid high copper-containing foods. The effectiveness of the treatment was assessed by clinical resolution of symptoms and serial determination of the liver enzymes, coagulation profile, serum albumin, and serum bilirubin (Table 1).

**Figure 2:**
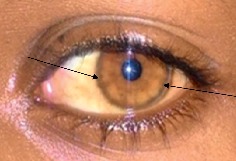
K-F ring on follow-up, which was after zinc treatment and has significantly decreased from the initial size of pigmentation.

On follow-up, our patient started to improve clinically (the symptoms and signs disappeared), and the deranged laboratory findings also improved initially (Table 1). Finally, the patient was discharged with zinc acetate (25 mg PO TID) and low copper diet (the patient was given a written paper in the local language of what food to avoid). Our patient is still on follow-up in one of the pediatric clinics (GI-clinic) with normal laboratory findings and free of clinical symptoms. He also had an improvement in the Keyser–Fleischer ring both clinically (Figure 2) and on slit examination. On follow-up, the family ( two younger siblings and his parents) were screened with serum ceruloplasmin, and his mother had a low level of ceruloplasmin, but she is asymptomatic.

## 2. Discussion and Conclusion

WD is rarely diagnosed and reported in African countries except in North African countries. In North African countries where consanguinity is common, autosomal recessive disease, including Wilson disease, is relatively common and reported. There are also reports from Senegal and Nigeria (two case reports). But the exact epidemiology of WD in Africa is not known because it is rare to diagnose and report. In those patients with signs and symptoms of liver disease which is not due to viral infections, WD must be considered and investigated because hepatic manifestations are common in children [4–7]. The diagnosis of WD relies on clinical manifestations like the pigmentation of the iris (Kayser–Fleischer rings, suggestive signs and symptoms of liver disease, and neuropsychiatric problems) with some laboratory pieces of evidence (low ceruloplasmin, elevated urine, and hepatic copper level) [5, 6]. However, the presence of Keyser–Fleischer rings and a low level of ceruloplasmin is sufficient to diagnose WD, especially in developing countries with limited resources [7]. Hepatic manifestation is more common in children with the symptoms ranging from an asymptomatic elevation of liver enzymes to a fulminant course [1, 8, 9]. So, in any patient with an unexplained liver disease, the disease must be investigated for possible WD and relatives of WD patients must also be screened for any asymptomatic WD with ceruloplasmin or urine copper, so that they will start early management [7].

Our patient had signs and symptoms of liver disease (jaundice, peripheral edema, and ascites) with elevated liver enzymes, hypoalbuminemia, hyperbilirubinemia, and coagulation abnormality. He had Kyser–Fleischer ring in both clinical (Figure 1) and slit-lamp examination. In addition to the K–F ring, our patient had laboratory evidence (high urinary copper and low ceruloplasmin) for WD. Laboratory tests, including alkaline phosphatase, bilirubin, and serum aminotransferases, were elevated. Medical therapy is effective, but WD is not yet curable, and it is progressive and fatal if not diagnosed and treated early [1, 10]. The American Association for the Study of Liver Diseases (AASLD) and the European Association for the Study of the Liver (EASL) recommend the use of a copper chelating agent, such as D-penicillamine or trientine, in the initial treatment of symptomatic patients. Zinc therapy can be initiated in those presymptomatic patients or for maintenance after chelators in symptomatic patients [10–13]. Till now, different guidelines, including the standard Nelson Textbook of Pediatrics, recommend that all symptomatic patients with Wilson disease receive a chelating agent [11, 13–15]. But chelators have many side effects with limited access, and most patients with neurologic WD symptoms may exacerbate as high as 50% and some may be intolerant [11, 15]. Although zinc is less effective than chelators in established Wilson disease treatment, data are limited and uncontrolled. While zinc treatment is effective in neurologic WD and in asymptomatic patients, caution is needed in hepatic WD as there are reports of hepatic deterioration. This may be related to less efficient decoppering and any adverse effects of zinc with most common gastrointestinal irritations [14]. Thus, exclusive monotherapy with zinc in those with symptomatic WD is controversial [14, 15]. In cases of hepatic presentation, treatment is usually initiated with potentially toxic copper chelators (D-penicillamine or trientine). Although multiple studies have introduced zinc as less toxic, available in low cost for WD treatment, its use has been limited to adjunctive or single-agent maintenance options for asymptomatic patients [10]. From most observational studies, it has been found that zinc therapy is the best choice in presymptomatic patients as it is effective and has fewer adverse effects. Especially acutely ill hepatic patients might do better on D-penicillamine, as zinc acts too slowly in these patients [12]. The use of zinc monotherapy in patients suffering from decompensated liver disease has not been well documented with the controversial results, but there are few cases reported with successful treatment [10].

Milanino et al. used a 2-year course of zinc sulfate in the pediatric case and noted normalization of the liver function, negative copper balance, and reduction of inflammatory infiltration, necrosis, and fat deposition in the liver histology [16, 17]. Brewer et al. briefly commented on a patient presenting with hepatic failure who experienced marked improvement in hypoalbuminemia and resolution of ascites after 8 months of zinc acetate therapy after the initial 4 weeks of Trenton therapy. In the same study, the investigators reported on the histologic evaluation of consecutive liver biopsy examination in 7 patients undergoing zinc monotherapy. The pathologic review showed no progression of cirrhosis and in two cases showed a complete resolution of cirrhosis [18]. There was one case of a 58-year-old male patient reported in Virginia, admitted with acute liver failure caused by WD, who was initiated with zinc monotherapy while awaiting liver transplantation. Over a 1-year period with zinc monotherapy, the patient experienced normalization of hepatic synthetic function and resolution of hypoalbuminemia and coagulopathy. Clinical stabilization of variceal bleeding, ascites and lower-extremity edema was also observed. The patient was not a candidate for transplantation because of the improvement in symptoms and the stage of the disease which is assessed by the Child—Turcotte—Pugh score [10]. This was supported by a study done in Italy, which showed that zinc monotherapy is effective in controlling WD-related liver disease, both as first-line and as maintenance treatment in patients with milder liver disease diagnosed in childhood [18]. There was also a case report from Nigeria, an 8-year-old male patient, with decompensated liver disease with neurologic manifestations managed with zinc monotherapy because of the unavailability of copper chelator drugs, and the patient showed significant improvement in the clinical manifestations and laboratory abnormalities [6]. There are a few examples (Table 2) that summarize the improvement of decompensated liver on zinc treatment. In our current case report, there is documentation of clinical resolution and normalization of laboratory liver synthetic dysfunction with zinc treatment. There were no documented adverse effects on the time frame. So, zinc is less toxic, accessible, and inexpensive. This may give hope for countries with limited resources. And this example and other case reports hopefully will encourage future investigations on the monotherapeutic administration of zinc for symptomatic hepatic Wilson disease. This is especially important for developing countries which have no access to copper chelator drugs and liver transplantation options.

## 3. Strengths and Limitations

The strengths of this case report are that the patient fulfills the diagnostic criteria, there was no doubt about the diagnosis and the synthetic functions done, and the severity of liver dysfunction was also assessed. The patient is followed up until now in clinics, and the clinical and laboratory resolution of symptoms was assessed fully. Lack of diagnostic modality for liver biopsy and the genetic analysis might be considered as a limitation in this case report.

## Figures and Tables

**Table 1:** Laboratory result at presentation and on follow-up.

Laboratory parameters	At presentation	Subsequent laboratory measurement on follow-up			
		2^nd^ week	3^rd^ week	5^th^ week	7^th^ week
WBC (4.0–10.5 × 10^3^/L)	8.4				
Hemoglobin (12.5–16.1)	12				
Platelet (150–4500 × 10^3^)	247				
AST (5–45 IU/L)	130	97	59	30	37
ALT (5–45 IU/L)	58I	160	60	24	30
ALP (115 IU/L)	300				
Serum albumin (3.2–5.1 gm/L)	1.7	2.7	2.2	3.9	4
Total protein		9.3	8.4	8.5	8.7
PT (11–14 sec)	28.8	32.4	32	18.1	12
APTT (25–35 sec)	38.5	36.8	35	34.2	30
INR (0.8–1.2)	2.62	2.88	2.8	1.62	1.3
Total bilirubin (mg/dl)	5	5.3	3.2	0.8	1
Serum ceruloplasmin (20–60 mg/dl)	<8				
24 hr urine copper (mcg/dl)	150				
Slit lamp examination	Kayser-Fleischer rings				

**Table 2:** Summary of improved decompensated liver disease after zinc treatment.

No	Sex	Diagnosis	Country	Treatment	Publication details	Year of publication
1	M	Decompensated liver disease with CNS	Nigeria	Zinc	Christopher I., et al.; Wilson disease in a Nigerian child: a case report; BMJ	2012
2	F	Liver disease with CNS	Nigeria	Zinc	Longe A. C., Glew R. H., Omene J. A; Wilson's disease. Report of a case in a Nigerian; Arch neurol	1892
3	F	Decompensated liver disease	Virginia	Zinc	Vanessa D. L., Patrick G. N., and Carl L. B.; Resolution of decompensated cirrhosis from Wilson's disease with zinc monotherapy: a potential therapeutic option; Clinical Gastroenterology and Hepatology	2006
4	M	Decompensated liver disease	Italy	Zinc	Brewer G. J., et al.; Treatment of Wilson's disease with zinc: initial treatment studies; Clinical MED	1989

5	Twenty-two children with Wilson disease with different degree of liver disease treated and followed up for 10 years with zinc acetate, and they show liver function and symptom improvement with no complications.				J Lab Clin Med.; 145(3):139–43	2005
6	Zinc monotherapy is effective in 44 Wilson disease patients, with mild liver disease diagnosed in childhood:				Ranucci et al; Orphanet Journal of Rare Diseases, 9:41	2014

## Abbreviations

WD: Wilson disease
ALF: Acute liver failure
K-F ring: Keyser-Fleischer ring
AST: Aspartate aminotransferase
ALT: Alanine aminotransferase
ALP: Alkaline phosphatase.

## References

[1] Ala A., Walker A. P., Ashkan K., Dooley J. S., Schilsky M. L. (2007). Wilson’s disease. *The Lancet*.

[2] Qin-Yun D., Zhi-Ying W. (2012). Advance in the pathogenesis and treatment of Wilson disease. *Translational Neurodegeneration*.

[3] Patil M., Sheth K. A., Krishnamurthy A. C., Devarbhavi H. (2013). A review and current perspective on Wilson disease. *Journal of Clinical and Experimental Hepatology*.

[4] More K. S., Dee Park K., Lalit C. (2014). Wilson’s disease; a rare presentation, a case report. *Medpulse International Medical Journal*.

[5] Ella W., Orit P., Peretz (2014). Late-onset fulminant Wilson’s disease: a case report and review of the literature. *World Journal of Gastroenterology*.

[6] Esezobor C. I., Banjoko N., Rotimi-Samuel A., Afolabi Lesi F. E. (2012). Wilson disease in a Nigerian child: a case report. *Journal of Medical Case Reports*.

[7] Schilsky M. L. (2002). Diagnosis and treatment of Wilson’s disease. *Pediatric Transplantation*.

[8] Korman J. D., Volenberg I., Balko J. (2008). Screening for Wilson disease in acute liver failure: a comparison of currently available diagnostic test. *Hepatology*.

[9] Aydinli M., Harmanci O., Ersoy O. (2006). Two unusual cases with Wilson’s disease: hepatoma and fulminant hepatitis treated with plasma exchange. *Journal of the National Medical Association*.

[10] Vanessa D. L., Patrick G. N., Carl L. B. (2006). Resolution of decompensated cirrhosis from Wilson’s disease with zinc monotherapy: a potential therapeutic option. *Clinical Gastroenterology, and Hepatology*.

[11] Hill G. M., Brewer G. J., Prasad A. S., Hydrick C. R., Hartmann D. E. (1987). Treatment of Wilson’s disease with zinc; oral zinc therapy regimens. *Hepatology*.

[12] Wiggelinkhuizen M., Talinus M. E. C., Bollen C. W., Houwen R. H. J. (2009). Systemic review: clinical efficacy of chelator agents and zinc in the initial treatment of Wilson disease. *Alimentary Pharmacology & Therapeutics*.

[13] European Association for the Study of the Liver (2012). EASL clinical practice guidelines: Wilson’s disease. *Journal of Hepatology*.

[14] Santiago R., Gottrand F., Debray D. (2015). Zinc therapy for wilson disease in children in french pediatric centers. *Journal of Pediatric Gastroenterology and Nutrition*.

[15] Balistreri W. F., Carey R. G. (2018). Metabolic Diseases of the Liver. *Nelson Textbook of Pediatrics*.

[16] Milanino R., Deganello A., Marrella M. (1992). Oral zinc as initial therapy in Wilson’s disease: two years of continuous treatment in a 10-year-old child. *Acta Paediatrica*.

[17] Milanino R., Marrella M., Moretti U. (1989). Oral zinc sulphate as primary therapeutic intervention in a child with Wilson disease. *European Journal of Pediatrics*.

[18] Brewer G. J., Yuzbasiyan-Gurkan V., Lee D. Y., Appelman H. (1989). Treatment of Wilson’s disease with zinc: initial treatment studies. *Journal of Clinical Medicine*.

